# Confirmation of co-denitrification in grazed grassland

**DOI:** 10.1038/srep17361

**Published:** 2015-11-30

**Authors:** Diana R. Selbie, Gary J. Lanigan, Ronald J. Laughlin, Hong J. Di, James L. Moir, Keith C. Cameron, Tim J. Clough, Catherine J. Watson, James Grant, Cathal Somers, Karl G. Richards

**Affiliations:** 1 Teagasc, Johnstown Castle, Environmental Research Centre, County Wexford, Ireland; 2 AgResearch, Ruakura Research Centre, Hamilton, New Zealand; 3 Soil & Physical Sciences Department, Lincoln University, Christchurch, New Zealand; 4 Agri-Environment Branch, Agri-Food & Biosciences Institute, Belfast BT9 5PX, UK; 5 Statistics and Applied Physics, Teagasc, Ashtown, Dublin 15, Ireland

## Abstract

Pasture-based livestock systems are often associated with losses of reactive forms of nitrogen (N) to the environment. Research has focused on losses to air and water due to the health, economic and environmental impacts of reactive N. Di-nitrogen (N_2_) emissions are still poorly characterized, both in terms of the processes involved and their magnitude, due to financial and methodological constraints. Relatively few studies have focused on quantifying N_2_ losses *in vivo* and fewer still have examined the relative contribution of the different N_2_ emission processes, particularly in grazed pastures. We used a combination of a high ^15^N isotopic enrichment of applied N with a high precision of determination of ^15^N isotopic enrichment by isotope-ratio mass spectrometry to measure N_2_ emissions in the field. We report that 55.8 g N m^−2^ (95%, CI 38 to 77 g m^−2^) was emitted as N_2_ by the process of co-denitrification in pastoral soils over 123 days following urine deposition (100 g N m^−2^), compared to only 1.1 g N m^−2^ (0.4 to 2.8 g m^−2^) from denitrification. This study provides strong evidence for co-denitrification as a major N_2_ production pathway, which has significant implications for understanding the N budgets of pastoral ecosystems.

It has been estimated that at the beginning of the 21^st^ century almost half the global population depended on fertilizer nitrogen (N) for its food supply^1^. Global population growth is predicted to further increase the demand for food by up to 100% by 2050^2^ and there is a need to meet this in an environmentally and economically sustainable manner^3^. More specifically, the global demand for meat and dairy products is predicted to increase by over 30%, driven by increased affluence in the developing world^4^. Pasture-based livestock systems account for 25% of global land area and are inherently ‘leaky’ in terms of N, with less than 30% of the applied N recovered in milk and meat products^5^. Applying current farming methods to meet increased global food demands is thus likely to result in a further acceleration of the N cycle, due to increased fertilizer use and deposition of animal excreta^6^. Full recovery of applied N in grassland remains elusive, with 20–40% of applied N often unaccounted for^7^,^8^,^9^ because soil N transformations result in the formation of reactive N (N_r_) compounds which include nitrate (NO_3_^−^), ammonia (NH_3_) and nitrous oxide (N_2_O). Globally, livestock are responsible for 65% of N_2_O emissions, 64% of NH_3_ emissions and 60% of leached N, with animal excreta being the principal source^10^,^11^,^12^. In grazed pasture systems most N_r_ losses arise from N deposited in the form of ruminant urine, which results in localized increases in N loadings ranging from 20 to 120 g N m^−2^ and which generally exceed the pasture plants’ immediate requirements^11^.

Loss of N_r_ from grazed pasture systems occurs via inorganic N leaching and overland flow to surface and ground waters, NH_3_ volatilization, and emissions of both N_2_O and di-nitrogen (N_2_) via biotic or abiotic mechanisms^9^. Nitrogen loss as N_2_, while potentially unacceptable on an economic basis, completes the N cycle and returns N to the atmosphere in an environmentally benign form. The loss of N_2_ from pasture livestock systems is not nearly as well studied as Nr losses. However, a more complete understanding of factors influencing N_2_ emissions could further elucidate Nr loss pathways. Isotopic mass balance studies have consistently failed to account for 20% of applied N^9^,^13^, with N_2_ emissions assumed to be the main source of uncertainty.

A commonly used methodology for calculating N_2_ and N_2_O fluxes arising from denitrification in ^15^N balance studies is that of Mulvaney and Boast^14^, which assumes that ^14^N and ^15^N atoms are randomly distributed during generation of the gas of interest (N_2_ or N_2_O) and that the NO_3_^−^ pool, from which N_2_ is derived, is isotopically uniform. If these assumptions are violated the gas flux may be underestimated^15^,^16^.

Di-nitrogen is the end product of conventional or ‘true’ denitrification, also referred to as canonical denitrification, in which NO_3_^−^ is sequentially reduced, via obligate intermediaries: NO_2_^−^, NO, and N_2_O. Denitrification is mediated by a range of microorganisms and occurs under anoxic or hypoxic conditions^17^,^18^. Nitrifying organisms may also produce N_2_O and N_2_ under reduced oxygen conditions in a process known as nitrifier-denitrification while true nitrification only results in N_2_O emissions^18^,^19^.

A process rarely considered in determining gaseous contributions to ^15^N mass balances is that of co-denitrification. Whilst hybrid N_2_ production is recognized in microbiology^20^, it has seldom been quantified in soil N process studies. Co-denitrification produces N_2_O (N_2_O_CO_) and N_2_ (N_2CO_) when, during sequential binding, a side reaction occurs between the initial electrophilic enzyme/N species complex and a nucleophile^21^,^22^. As a result, co-denitrification results in hybrid N_2_ and/or N_2_O molecules that are formed from isotopically non-uniform pools, with one N atom of NO/NO_2_ derived from an inorganic N source (NO_3_^−^, NO_2_^−^ or NO^−^) and another nucleophilic N atom from a co-substrate^17^,^22^ (usually N_3_, NH_3_ or a monomeric organic N source such as an amine) (Fig. 1). Whereas abiotic N_2_ production has been shown to occur at low pH (<5.2)^23^,^24^, N_2_ from co-denitrification is recognized as a biotic process occurring under intermediate to high pH conditions (>6)^22^.

In grazed pastures N input from ruminant urine is the main source of N cycling and loss^25^. Subsequent N transformations within the created urine patch, including elevated pH and inorganic N concentrations, and the stimulated microbial activity, drive Nr losses^26^,^27^,^28^. Isotopic mass balance studies have consistently failed to account for 20% of applied N^9^,^13^, with N_2_ emissions assumed to be the main source of uncertainty. Potentially, this is because the contribution of co-denitrification to gaseous N losses from ruminant urine has not yet been investigated under intensively managed grasslands.

In a previous laboratory study, N_2_ and N_2_O emissions accounted for 30–65% and <5%, respectively, of the urine N applied to an undisturbed grassland soil over a 30-day period^29^ but the authors were unable to identify the specific processes contributing to the high N_2_ emissions. Identification of such processes would enable a better understanding of N use efficiency in agricultural systems. This paper presents a novel approach that combines two ^15^N flux determination methodologies in order to differentiate the relative contributions of true denitrification and co-denitrification^14^,^23^,^30^.

Therefore, the objective of this paper was to determine the relative contributions of the denitrification and co-denitrification processes to N_2_ emissions from ruminant urine applied to a pastoral soil.

## Results and Discussion

Denitrification of ^15^N-labelled N pools results in the generation of N_2_ gas where either one or both N atoms are ^15^N-labelled, giving mass numbers 29 or 30, respectively. Co-denitrification results in the formation of hybrid N_2_ where a large proportion of N_2_ molecules are produced as ^29^N, relative to ^30^N, and the ratio of Δ^29^R to Δ^30^R, derived solely from co-denitrification, will be 272^23^ (see SI methods for further details). The observed mean ratio of Δ^29^R / Δ^30^R in this study was 214, indicating a substantial contribution of co-denitrification to the total N_2_ efflux. Using the conventional equations of Mulvaney and Boast^14^, the overall mean ^15^N enrichment of the pool from which the N_2_ was derived (^15^X_N_) could be quantified, and this was calculated to be 0.0214 (2.14 atom% ^15^N). If true denitrification was occurring, this ^15^X_N_ value should have been similar to the ^15^N enrichment of the pool from which N_2_O was derived (N_2_O_aD_). However, this was not the case, as the mean N_2_O_aD_ value was calculated to be 36 atom% ^15^N, a far greater enrichment than that of the calculated ^15^X_N_. Hence a process other than true denitrification was responsible for the majority of the N_2_ produced.

It is unlikely that anaerobic ammonium oxidation (ANAMMOX) was responsible for the high N_2_ emissions, although its contribution cannot be ruled out. While ANAMMOX is recognised as a significant N_2_ production mechanism in aquatic systems^31^,^32^, its contribution to N_2_ production in terrestrial biomes has to date been recognised only in rice paddy soils and wetland soils^32^,^33^, characterised by anoxic conditions. Evidence to date suggests the contribution of ANAMMOX to N_2_ production from soils might be low, given that the ANAMMOX-specific genetic potential has also been shown to be low in wetland soils^33^. Trimmer and Purdy^34^ found an uncharacterised metabolism potentially capable of oxidising organic-N (e.g. NH_2_ groups) directly to N_2_, a process that was neither ANAMMOX nor denitrification. We suggest that the process Trimmer and Purdy observed was most likely co-denitrification. Brabandere *et al.*^35^ discussed how their experiment may have underestimated ANAMMOX and they suggest that the direct use of amines from dissolved organic N in a process similar to ANAMMOX may be responsible, but that this process has not yet been linked to a specific microbial metabolism. Our paper is the first time an attempt has been made to assess co-denitrification in a pastoral ecosystem where it plays a major role and may be the reason why circa 20% of N is still unaccounted for in N mass balances.

**Figure 2:**
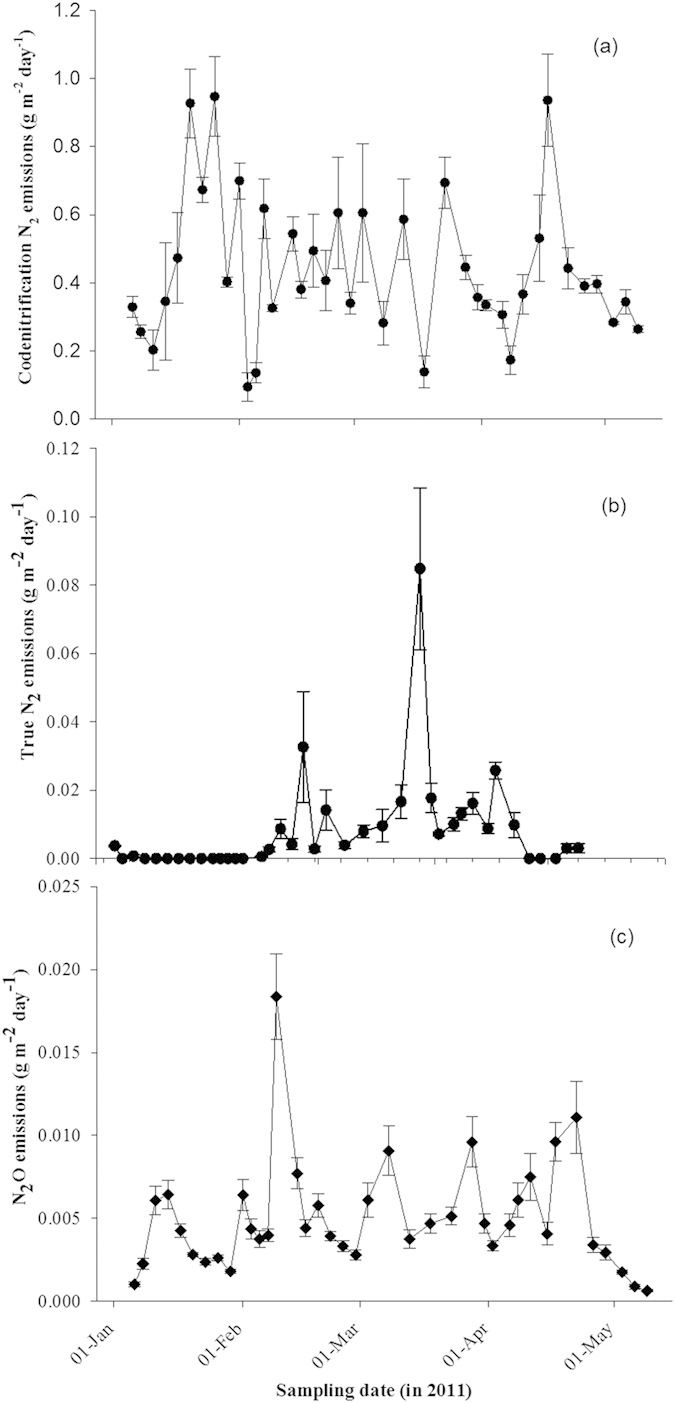
Mean daily N_2_ and N_2_O fluxes (g N m^−2^ day^−1^) (n = 4) from (**a**) co-denitrification (N_2CO_), and true denitrification (**b**) N_2TRUE_, and (**c**) N_2_O_TRUE_ over a four month period following urine deposition. Error bar is the standard error of the mean (n = 4).

**Figure 3:**
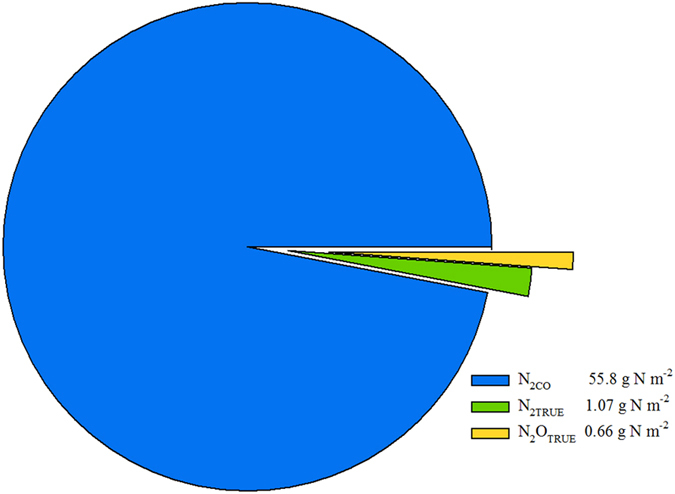
Cumulative N_2_ and N_2_O emissions from co-denitrification (N_2CO_), and true denitrification (N_2TRUE_ and N_2_O_TRUE_) over a four month period following urine deposition. Confidence intervals (95%) (n = 4) from analysis of the treatments, were 38 to 77 g m^-2^ for N_2CO_, 0.4 to 2.8 g m^-2^ for N_2TRUE_, and 0.27 to 0.77 g m^-2^ for N_2_O_TRUE_.

Temporal profiles of gaseous N emissions revealed substantial losses associated with N_2CO_, with mean daily fluxes of 0.44 g N m^−2^ d^−1^ over the four-month experiment (Fig. 2a). In contrast, emissions associated with true denitrification were an order of magnitude lower; with mean daily fluxes of 0.01 g N m^−2^ d^−1^ and 0.005 g N m^−2^ d^−1^ observed for N_2TRUE_ and N_2_O_TRUE_, respectively (Fig. 2b,c). Although N_2CO_ was the predominant loss pathway there was no detectable N_2_O_CO_ during the course of the experiment. The cumulative gaseous N losses associated with true denitrification were 1.1 and 0.66 g N m^−2^ for N_2TRUE_ and N_2_O_TRUE_, respectively (Fig. 3). Emissions from N_2CO_ were the dominant loss pathway accounting for 55.8 g N m^−2^ and 97% of total gaseous N loss, equivalent to 56% of the N applied. Comparisons with prior studies are limited due to the fact that only one laboratory study reports co-denitrification, to our knowledge^36^. Their study^36^ showed that 92% of the N_2_ emitted was due to co-denitrification and only 8% due to denitrification after applying ^15^N enriched NH_4_NO_3_. These findings are similar to the findings in the current study.

**Figure 4:**
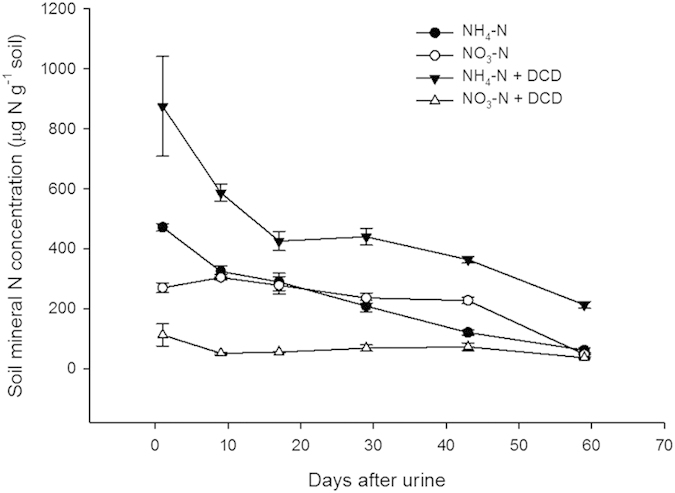
The effect of the nitrification inhibitor DCD on soil ammonium and nitrate concentrations in urine-affected soil, measured from aligned small plots receiving the same treatments as lysimeters in the main study. Error bar is the standard error of the mean (n = 4).

**Figure 5:**
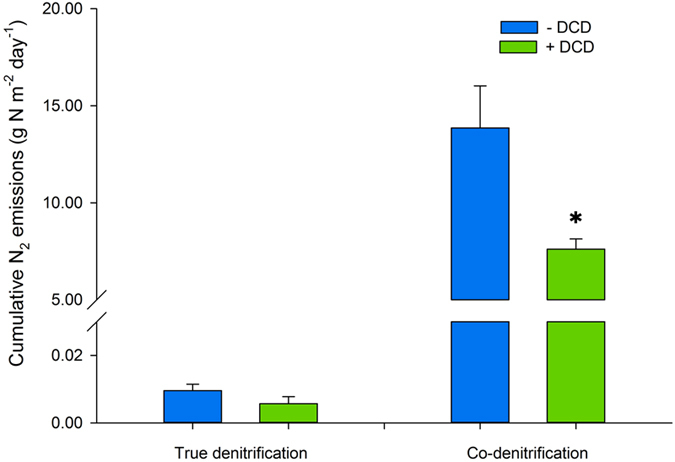
Effect of the nitrification inhibitor DCD on cumulative N_2_ emissions from true denitrification (N_2TRUE_) and co-denitrification (N_2CO_) (g N m^-2^) over the first month following urine deposition. Significant differences between “+DCD” and “−DCD” for each process are marked with *(P < 0.05). Error bar is the standard error of the mean (n = 4).

In order to investigate the underlying drivers of the N_2_ emissions, we assessed the effect of nitrification on both N_2TRUE_ and N_2CO_, using the nitrification inhibitor dicyandiamide (DCD) which was applied within a subset of ^15^N-labelled urine treatments. Incorporating DCD with the urine inhibited the first stage of nitrification, the oxidation of NH_4_^+^ to NO_2_^−^ (Fig. 4). Over the first 30 days of the experiment, when the nitrification inhibition was effective, cumulative N_2_ emissions were significantly (*P* < 0.05) reduced by 55% for N_2CO_ and no significant changes were found for N_2TRUE_ (Fig. 5). This indicates that reduced NO_2_^−^ formation, resulting from nitrification inhibition, affected N_2CO_. This finding, combined with the significantly higher N_2CO_ contribution to the N_2_ flux than from N_2TRUE_ (Fig. 3), indicates that different N pools supplied each process. The reduced nitrification activity in the presence of DCD and associated reduction in N_2CO_ flux demonstrates that one source pool for co-denitrification (the applied N contribution) was the NO_2_^−^ produced during nitrification (oxidation of NH_4_^+^ to NO_2_^−^), rather than true denitrification (reduction of NO_3_^−^ to NO_2_^−^)^37^.

Urine N deposition on pastoral soils may provide the optimal conditions for co-denitrification, due to urine patches containing high inputs of N (mainly urea-N), localised regions of elevated soil pH, and because urine increases the supply and turnover of labile soil organic N and C^26^,^30^. The formation of both hybrid N_2_ and N_2_O has been shown to be promoted by increases in pH^38^,^39^. Urine patches represent localized areas of high pH (circa. 8–10) due to hydrolysis reactions^40^,^41^. See Supplementary Figure S1 online for pH under urine-affected soil.

During the second stage of nitrification, the oxidation of NO_2_^−^ to NO_3_^−^, can be inhibited by elevated pH and high ammonia-N concentrations^26^,^42^,^43^. We applied 1000 μg urea-N g soil^−1^, with previous studies having shown that 400 μg NH_4_^+^-N g soil^−1^ was sufficient to inhibit NO_2_^−^ oxidation^41^. Whereas, in unfertilized soils, NO_2_^−^ pools are very low (<5 μg NO_2_^−^-N g soil^−1^) as the oxidation of NH_4_^+^ to NO_2_^−^ proceeds at a slower rate than the subsequent oxidation of NO_2_^−^ to NO_3_^−^. In a controlled incubation study with the same soil type and urine N input (1000 μg urea-N g soil^−1^), soil pH was elevated from days 0–40, during which NH_4_^+^ concentrations peaked soon after urine addition then steadily declined; which confirms expected observations in urine-affected soil^27^. However, there was a delay in NO_2_^−^ or NO_3_^−^ produced from days 10–25 during which time NH_4_^+^ concentrations were declining, and soil pH remained > 6, suggesting a substantial loss of N.

Elevated soil pH under urine patches results in the hydrolysis of organic matter and increased microbial turnover of organic N and C^29^. Denitrification rates have been shown to be correlated with the availability of labile organic C for the supply of reductant^44^, and when combined with large pools of labile, nucleophilic organic N, this may result in the formation of hybrid (N-N linkage) denitrification end-products^22^. Indeed, this scenario has been hypothesized for both hybrid N_2_O and N_2_ formation observed under high C and elevated carbon dioxide (CO_2_) conditions^45^,^46^.

In the current study, N_2_ rather than N_2_O was the end product of co-denitrification. The reason for this is not readily apparent, but may be related to the presence of different microorganisms, cofactors such as copper and iron complexes, the form of metabolisable carbon substrates present, and the type and oxidative state of the alternative nucleophilic N substrates (e.g., hydrazine, ammonia, ethylene diamine, aniline and/or amino acids)^47^. Soil pH has also been shown to favour N_2_O reductase^48^ activity and therefore promote N_2_ formation, which suggests the possibility of conversion of hybrid N_2_O to hybrid N_2_ under urine-affected soil when the pH is >6, as in this study.

Examples of where soil microorganisms have influenced the ratio of N_2_O to N_2_ co-denitrification product ratio include that of Okada *et al.*^49^. Where *Mesorhizobium spp.* under oxic conditions were observed to produce only hybrid N_2_, with amino acid comprising the unlabelled N source. Grazed pastoral soils have been shown to have high free amino acid levels, accounting for 10–40% of the soluble N in soil solution^50^ and it is perhaps possible that co-denitrification products favour N_2_ formation as a consequence of the microbiology that exists. For example, soil fungi have been suggested to be the principal microbial source of N_2_ emissions from co-denitrification^36^,^51^.

The N_2CO_ losses of 55.8 g N m^−2^, equating to an N loss of 56% of the N applied (in mass balance terms), are greater than all other N loss pathways from the urine patch^25^. Interestingly, our finding of 56% of applied N lost as N_2_ fits within the range of loss (as a percentage of N applied) reported by Monaghan and Barraclough^29^, the only other direct measurement of N_2_ emissions from urine-affected soil we could find and within which the authors were unable to identify the process(es) responsible. Clough *et al.*^9^ estimated N_2_ loss of a similar magnitude to N_2TRUE_ measured in the current study which suggests that N_2_ emissions from co-denitrification may have been overlooked. These high rates of N_2CO_ also represent a substantial mobilization of native soil N from a urine patch (27.9 g N m^−2^), given that half the N_2CO_ is derived from the applied urine N and half from native soil N. Rather than indicating a priming effect or net loss of soil N, the contribution of native soil N to N_2_ emissions indicates substantial mineralization-immobilization turnover (MIT) beneath a urine patch^29^,^52^, over the short term (<12 months) rather than the long term^53^. Nevertheless, the underlying drivers for the removal of a substantial proportion of applied and native soil N requires further investigation.

## Methods:

### Experimental set-up

Intact monolith lysimeters, 0.5 m in diameter and 0.7 m deep, were collected from a permanent grazed grassland soil^54^ in the south-east of Ireland (8°15′W, 52°9′N, County Cork) and installed in the Johnstown Castle Environmental Research Centre lysimeter facility (52°17′N, 6°30′W) in County Wexford, Ireland. The area sampled had not been grazed or received manure applications for the previous eight weeks. The soil was a free-draining haplic cambisol^55^ which was typical of those used for dairy production. Total N was 0.42% and total carbon 4.2%. Cattle urine was collected in late autumn 2010 from mixed age Holstein-Friesian dairy cows during milking and analyzed for total N concentration. Urine-N was amended with 98 atom% ^15^N-labelled urea resulting in a urine N concentration of 10 g N L^−1^ and a ^15^N enrichment of 45 atom% excess. The authors confirm that all experiments were performed in accordance with relevant Teagasc guidelines and regulations and all experiments were approved by Teagasc.

Two liters of labelled urine were applied to each lysimeter on 28 December 2010 (late winter), simulating a urine patch in the field with an equivalent N loading rate of 1000 kg N ha^−1^. To understand the role of nitrification on N fluxes, dicyandiamide (DCD; Sigma Aldrich, Germany) was used to inhibit nitrification^56^. The DCD was sprayed in solution form (20 mL of 15 g DCD L^−1^ solution) onto the surface of the lysimeters in two split applications of 15 kg DCD ha^−1^, one immediately after urine application and again two months later. There were two treatments: (1) ^15^N-labelled urine (“no DCD”) and (2) ^15^N-labelled urine with DCD (“DCD”). There were four replicate lysimeters for each treatment. The climatic conditions during the 123 day experimental period 28 December 2010 to 9 May 2011, were typical for the area. The cumulative rainfall input was 241 mm, which was slightly lower than the long-term average for the area (January to April, inclusive) of 288 mm. The average maximum and minimum air temperatures were 10.5 and 4.9 °C, respectively, and the average daily air temperature was 7.8 °C, which was slightly higher than the long-term average of 6.8 °C. See Supplementary Figure S2 and S3 show estimated soil water-filled pore space (WFPS) from the lysimeters and daily average air and 100 mm depth soil temperature.

### Aligned soil plots

Intact monolith plots, 0.5 m in diameter and 0.2 m deep, were also collected from the same soil type, and received the same urine and DCD treatments as the lysimeters. There were four replicates per treatment. These plots were sampled for soil to 75 mm depth every 10 days (on average) during the first 60 days after treatment application. Soil samples were passed through a 4 mm sieve and extracted with 2 M potassium chloride (KCl) solution. The filtrate was analysed for NH_4_^+^-N and NO_3_^−^-N concentrations on an Aquakem 600 A automated analyzer (Thermo Electron, Sweden). Blank KCl samples were used to account for any residual N in the extracting solution and results were expressed as a concentration of N in the soil (μg N g^−1^ soil).

### Gas analysis

A static closed chamber method was used to measure N_2_O and N_2_ emissions^57^. During measurement the outside edge of the chamber was inserted into a water channel built around the top edge of the lysimeter casing in order to ensure a gas-tight seal. At the beginning of each measurement period the chamber was placed slowly on top of each lysimeter and headspace gas samples were taken through a butyl rubber septum fitted into the top of the chamber initially (t0), after 20 minutes (t20), 40 minutes (t40) and 120 (t120) minutes. Samples were transferred from the chamber to a pre-evacuated 7 ml glass vial using a plastic syringe. Headspace samples were taken between 12:00 and 15:00 hours. Samples were taken every 2 to 3 days (n = 39) for 123 days post urine application.

Headspace N_2_O concentration was quantified using gas chromatography (Varian 3800 GC with ECD detector). Headspace N_2_ and N_2_O samples were analyzed for ^15^N on a Thermo-Finnegan Delta-S isotope ratio mass spectrometry (IRMS) interfaced with a GC capillary column in the UC Davis Stable Isotope Facility (http://stableisotopefacility.ucdavis.edu). The limit of quantitation for the isotopic ratio mass spectrometry analysis of N_2_O and N_2_ was 150 ppmv (standard deviation of 0.1‰).

True denitrification and co-denitrification to N_2_ were calculated using the ^15^N flux method^36^. In brief, for N_2_O the ion currents (I) at *m/z* 44, 45, and 46 enabled concentrations and molecular ratios ^45^R (^45^I/^44^I) and ^46^R (^46^I/^44^I) to be calculated. The sources of N_2_O were then apportioned into the fraction (*d’*_*D*_) derived from the denitrifying pool of enrichment a_D_ and the fraction *d′*_*N*_ = (1 − *d′*_*D*_) derived from the pool or pools at natural abundance^58^. For N_2_, the ion currents at *m/z* 28, 29 and 30 enabled molecular ratios ^29^R (^29^I/^28^I) and ^30^R (^30^I/^28^I) to be determined. Differences between the molecular ratios of enriched and ambient atmospheres were expressed as Δ^29^R and Δ^30^R. The flux of N_2_ was calculated using three different methods:

1. Δ^29^R and Δ^30^R were used to calculate the enrichment of the denitrifying pool (^15^X_N_) and then the N_2_ flux according to Mulvaney and Boast^14^;
2. using Δ^30^R data only and the equation of Mulvaney^59^ assuming that the enrichment of the denitrifying pool was a_D_^30^; and
3. using Δ^29^R and Δ^30^R to calculate a separate contribution due to co-denitrification (N_2CO_) and true denitrification (N_2TRUE_) calculated by Method 2.

True denitrification contributes to Δ^29^R and Δ^30^R whereas co-denitrification contributes mostly to Δ^29^R, the Δ^29^R to Δ^30^R ratio always being 272^23^. All of the Δ^30^R was assumed to be derived from true denitrification, so Δ^30^R was used to calculate the flux of N_2_ due to denitrification by Method 2. Using the ‘backsolver’ facility in Microsoft Excel^™^, the value of Δ^29^R that could be attributed to N_2TRUE_ was then obtained. The difference between the total measured Δ^29^R and Δ^29^R due to true denitrification was assigned to co-denitrification. The fraction of the total moles of N_2_ in the headspace from co-denitrification (*d*_CD_) was calculated using Equation 1^23^.


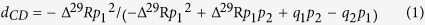


where *p*_1_ (0.9963) and *q*_1_ (0.0037) are atom fractions of ^14^N and ^15^N in the natural abundance pool; *p*_2_ and *q*_2_ are the atom fractions of ^14^N and ^15^N in the enriched NO_3_^−^ pool from which co-denitrification is assumed to occur. To determine the rates of N_2TRUE_ and N_2CO_, the total air volume of the chamber, corrected for standard temperature and pressure, was calculated and then the amount of N_2_-N in the headspace determined. The amount of N_2_-N derived from true denitrification and co-denitrification was determined by multiplying the total amount of N_2_-N in the headspace by *d* and *d*_CD_, respectively. Denitrification rates were expressed as g N m^−2^ day^−1^.

### Statistical analysis

Data analysis was conducted on cumulative fluxes from the full 123 day observation period and from the first month following urine application (to 1-Feb), to test for a DCD treatment effect. Confidence intervals for the final cumulative figures were obtained from the fitted model for the analysis of cumulative emissions. These were conservative estimates as they were based on treatment outcomes (n = 4). Log transformation was used as required to approximate constant variance and normally distributed data. Means from analyses on the log scale, with their confidence intervals, were back-transformed for interpretation on the original data scale and presentation in figures and text.

The analysis of the cumulative data was a t-test of the outcomes from four replicate lysimeters per treatment. The analysis model was fitted with the linear model procedures of the SAS statistical package (SAS Institute Inc., Cary, NC). Residual checks were made to ensure that the assumptions of the analysis were not violated. Influence statistics (e.g. Cook’s D, Restricted Likelihood Distance) were used to check whether one or more points could be creating bias in the analysis outcome.

The cumulative N-gas emissions were calculated by linear interpolation between sampling days and trapezoidal calculation^60^. Seventeen of the 312 observations were removed where the N_2_O concentration was below the IRMS limit of detection or where there were excessively high N_2_ enrichment readings, which caused numerical errors to propagate through the calculations of the response resulting in non-credible outcomes. For the comparisons of a treatment effect in the first month (to 1-Feb) following urine deposition, only one lysimeter had a data point removed. Removal of that data point from the analysis did not affect the finding of significant difference between the treatments.

In order to test the scale of possible errors in total cumulative fluxes due to the removal of the data, the maximum and minimum observed values for the each of the affected lysimeters were substituted for the missing values. The corresponding maximum and minimum cumulative fluxes did not alter the general conclusion based on the magnitude of the cumulative N_2CO_ flux.

## Conclusions and Implications

We conclude that co-denitrification was the dominant process producing N_2_ beneath a urine patch, accounting for 97% of all denitrification-derived gaseous N_2_ loss. Previously published values of gaseous N emissions from grazed pasture systems which only accounted for emissions of N_2_O and N_2_ via conventional denitrification may have grossly underestimated the total N loss. Other workers have shown that either bacterial or fungal co-denitrification can occur in aerobic grassland soils. We contend that co-denitrification is likely to be a ubiquitous loss pathway in grazed grasslands but the magnitude of losses may be dependent on soil factors such as soil pH and organic matter content.

The quantification of significant N loss via co-denitrification has major environmental and economic implications. The large N surpluses identified in grazed grassland systems are often assumed to be lost as environmentally-damaging reactive N emissions. However, this may not always be the case as co-denitrification resulted in large, non-reactive N_2_ fluxes with dual contributions from the applied urine and native soil N, whereas reactive N_2_O fluxes were minimal. Nevertheless, the overwhelming size of the flux indicates that this loss of N, whilst environmentally benign, represents a considerable economic loss of soil N required for production of agricultural goods. Further studies on co-denitrification and the factors affecting its magnitude will help us to close the ‘gap’ in N budgets and improve N use efficiency in grazed grassland systems and perhaps in other terrestrial ecosystems on a global scale.

## Additional Information

**How to cite this article**: Selbie, D. R. *et al.* Confirmation of co-denitrification in grazed grassland. *Sci. Rep.* **5**, 17361; doi: 10.1038/srep17361 (2015).

## Supplementary Material

Supplementary Information

## Figures and Tables

**Figure 1:**
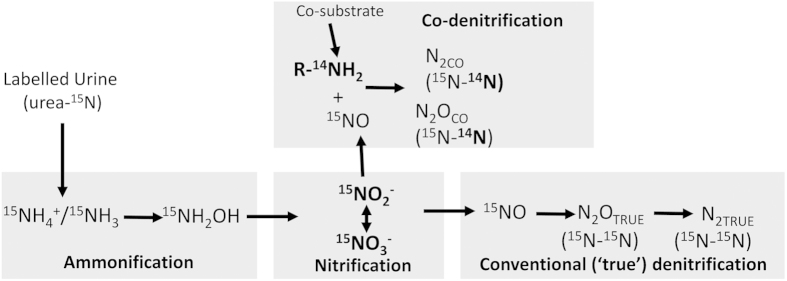
Conceptual model of co-denitrification under urine patches in grassland soils, commencing with urea, the dominant N substrate found in ruminant urine.
