# Supplementary material for: Confirmation of co-denitrification in grazed grassland
Source: Sci Rep. 2015 Nov 30;5:17361. doi: 10.1038/srep17361 (PMC4663629; doi:10.1038/srep17361)
Supplement: Supplementary Information [file srep17361-s1.doc]

**Supplementary Information**

**Title:**

Confirmation of co-denitrification in grazed grassland

**Authors:** Diana R. Selbie^1,2,3^, Gary J. Lanigan^1^, Ronald J. Laughlin^4^, Hong J. Di^3^, James L. Moir^3^, Keith C. Cameron^3^, Tim J. Clough^3^, Catherine J. Watson^4^, James Grant^5^, Cathal Somers^1^ & Karl G. Richards^1^*

**Affiliations:**

^1^ Teagasc, Johnstown Castle, Environmental Research Centre, County Wexford, Ireland.

^2^ AgResearch, Ruakura Research Centre, Hamilton, New Zealand.

^3^ Soil & Physical Sciences Department, Lincoln University, Christchurch, New Zealand.

^4^ Agri-Environment Branch, Agri-Food & Biosciences Institute, Belfast BT9 5PX, UK.

^5^ Statistics and Applied Physics, Teagasc, Ashtown, Dublin 15, Ireland.

*Corresponding author ([karl.richards@teagasc.ie](mailto:karl.richards@teagasc.ie))

**Keywords:**

Nitrogen cycle, co-denitrification, urine, grazed grassland, di-nitrogen, pastoral, N balance

Figure S1 | Concentrations of (a) ammonium-N, (b) nitrite-N, (c) nitrate-N and (d) soil pH (extracted with KCl), in an experiment conducted under controlled conditions on the same soil type, receiving 1000 µg urea-N g^-1^ soil (unpublished). Error bar is the standard error of the mean (n=4).

Figure S2 | Estimated water-filled pore space in the lysimeters during the experiment in 2011.

**Figure S3 | Daily average air temperature and 100 mm soil temperature estimated from a climate meteorological station adjacent to the lysimeter facility during the 123 day experimental period in 2011.**

**Additional description of the methods used to calculate the N_2_ flux**

The three calculation approaches, termed Methods 1, 2 and 3, used to estimate N_2_ production and the contributions from true and co-denitrification processes are described in detail by Laughlin and Stevens (36) (p.1541). Here, we provide an explanation for these methods within the context of the theory and advances in calculation methodology to date.

*Method 1: Δ^29^R and Δ^30^R were used to calculate the enrichment of the denitrifying pool (^15^X_N_) and d the proportion of N_2_ in the atmospheric sample (d) and then the N_2_ flux according to Mulvaney and Boast (14).*

This calculation uses Δ^29^R and Δ^30^R but does not take account of the fact that there is also a natural abundance pool, and assumes an enrichment of the chamber headspace in the *absence* of a natural abundance pool. From Mulvaney and Boast (14): “The equations used to perform these calculations are based on the assumption that the nitrate denitrified is isotopically uniform”.

Arah (58), Boast *et al.* (16) and ultimately Clough *et al.* (23) extend and refine this theory to account for the fact that a non-labelled (i.e. natural abundance) pool may contribute to N_2_O and N_2_ production. Arah (58) refined isotopic theory for triple detector IRMS instruments (that measured 29/28 and 30/28 ratios) and thus assumed that there is non-uniformity in the source pools of N. Figure S4 amended from Boast *et al.* (1988) visualizes this.


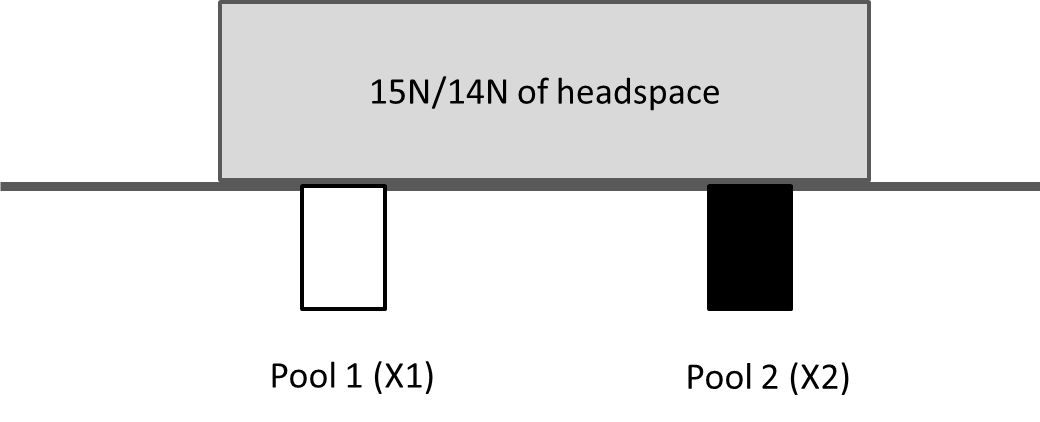


**Figure S4 | Schematic representation of non-uniformity in source pools of N.**

*Method 2: Using Δ^30^R data only and the equation of Mulvaney (59) assuming that the enrichment of the denitrifying pool was a_D_ (30).*

This equation accounts for N_2_ or N_2_O supplied from a single, uniformly labelled N pool (^15^N_2_ or ^15^N_2_O).

*Method 3: Using data for Δ^29^R and Δ^30^R to calculate a separate contribution because of co-denitrification in addition to N_2_ from denitrification calculated by Method 2.*

Co-denitrification is subsequently calculated from Δ^29^R and Δ^30^R. This separately calculates the contribution due to co-denitrification (N_2CO_) and true denitrification (N_2TRUE_) (Equation S1) from Mulvaney and Boast (14).

The equation is as follows:

=-(29/28R*(p1^2^))/(( -(29/28R) (p1^2^))+( 29/28R *p1p2)+(q1p2)-(q2p1)). Equation S1

This apportions 29/28 to labelled p2q2 and unlabelled p1q1 pools. A detailed derivation of the calculations for the contribution of co-denitrification to N_2_ production is provided as an appendix to Clough *et al.* (23).
